# The value co-creation mechanism of Public Cultural Services ecosystem in China based on "public value" theory

**DOI:** 10.1371/journal.pone.0315192

**Published:** 2025-01-15

**Authors:** Xianke Li, Johnny Fat Iam Lam, Zhicong Lin, Ke Song

**Affiliations:** 1 Faculty of Humanities and Social Sciences, Macao Polytechnic University, Macao, Macao; 2 Department of Public Administration, Party School of the Meizhou Committee of CPC, Meizhou, China; Kathmandu Model College, Tribhuvan University, NEPAL

## Abstract

The establishment of China’s public cultural service system has been explored for nearly two decades, but there is still a significant gap in achieving the goal of equalising and eliminating differences in public value, and it is urgent to systematically explore the public value governance issues contained in it. This paper presents a localised theoretical analysis framework based on the strategic triangle model of public value, with the objective of exploring the endogenous mechanism of China’s public cultural services ecosystem (PCSE). Furthermore, it constructs a panel vector autoregression (PVAR) model from a dynamic endogenous perspective, employing the STATA 16.0 software package to conduct empirical tests. The empirical testing of the interactions between government attention (GA), cultural needs (CN) and public cultural services (PCS), as well as the regional heterogeneity, is based on data from 31 provinces and municipalities in mainland China for the period of 2008–2022. The findings are as follows: (1) The unified analysis framework reveals a significant positive mutual promotion relationship between GA and PCS. In contrast, the two-way promotion relationship between CN and PCS is weak, while the relationship between GA and PCS is unstable. (2) The results of the dynamic response analysis demonstrate significant regional disparities in the value co-creation path of public cultural services. The circular transfer of CN and PCS is more pronounced in the eastern region, while the central-western regions rely more on the circular transfer of GA and PCS. This study offers insights for central and local governments to reinforce the value co-creation foundation of a service-oriented government, explore a value co-creation pathway tailored to local conditions, and construct a contemporary public cultural services ecosystem guided by the value of common prosperity in spiritual life.

## 1. Introduction

Modern theories of public management advocate for the government to provide higher quality public services, thereby entrusting the government with more functions in public management. With the continuous improvement of social administration systems and legal frameworks, as well as the integration of new technologies like the Internet into public service systems, there has been extensive participation from the public in supplying these services. This has led to a call for public managers to re-embrace their mission of serving the public [1]. It is of the utmost importance for public managers to reassess potential pitfalls related to instrumental rationality within their provision of services. In 2004, China officially introduced the concept of "building a service-oriented government," marking its full transition into a governance model suited for post-industrial societies [2]. Within this construction is an evolution from mere value creation to "serve the people" as the primary value orientation—surpassing the "serve the customer" in new public management [3]. Over the past two decades, the study of China’s modern governance system for public service delivery has become an important topic that has long attracted scholarly attention.

Since the 20th National Congress of the Communist Party of China, there has been a growing consensus among public administration departments and academia that the objective of common prosperity can be achieved through the high-quality development of public services. The demand for cultural services is an important indicator of the growing aspirations of the population for a better quality of life. The Chinese government has identified the contradiction between this demand and the unbalanced and inadequate development at the national level as a significant concern. In 2006, the Outline of the National Cultural Development Plan for the Eleventh Five-Year Plan Period proposed the incorporation of government public cultural services functions into its overall function system. Subsequently, in 2012, during the implementation phase of the "Twelfth Five-Year Plan", ideas and fundamental national standards were clarified in order to establish a comprehensive Public Cultural Services Ecosystem (PCSE) within the framework of the National Basic Public Service System. Furthermore, in 2021, the 14th Five-Year Plan for the Construction of the PCSE and Opinions on Promoting High-quality Development of the PCS were issued in succession. These documents represent an effort to advance the PCS towards becoming a higher-quality value co-creation system. The process has thus far yielded several unanswered questions. Such as, how to build an PCSE with government-led social participation? What is the effect of value co-creation? What about the co-creation path of public value?

Previous studies have provided significant implications for elucidating the value co-creation logic of the PCSE from various perspectives, such as system [4–6], community [7, 8], and symbiosis [9–12]. However, three key issues remain to be resolved. (1) Under the co-creation logic of public value, what is the mechanism of interaction between different stakeholders? The "government-led and social participation" implies that there may be compatible or incompatible interactions between multiple subjects. The majority of current studies focus on the connotations, fundamental characteristics, primary responsibilities, and promotional strategies of the contemporary PCSS under the necessity state [13–16]. However, there is a paucity of studies on the mechanism of co-creation of value under the reality state. (2) How can we establish a systematic analytical framework for value co-creation in the PCS? Value co-creation is a complex process, and therefore, the use of static measurement analysis of the PCS alone is inadequate for comprehending the holistic logic behind value co-creation [17, 18]. The key to addressing this issue lies in integrating the primary factors influencing the PCS’ value co-creation within a unified theoretical framework. And from the perspective of ecosystem, it is deeply analyzed whether the short-term deviation between them can be effectively corrected, so as to realize the dynamic evolution process of long-term equilibrium. (3) How can a support policy system be developed that is adaptable to local conditions within the framework of unified analysis? Ensuring equal access to basic the PCS is a crucial objective in co-creating public value. However, governments in different regions may vary significantly in their level of concern for cultural undertakings, demand for cultural services, and capacity to provide PCS. Moreover, the approaches towards co-creating value may also differ. It is therefore of great importance to explore ways in which carriers of the PCS across different regions can achieve these fundamental and equitable goals.

In response to the above questions, this paper first establishes a theoretical analysis framework for value co-creation in China’s PCSE. Subsequently, using panel data, this paper empirically tests the strategic triangular cycle relationship of value co-creation. Finally, targeted policy recommendations are proposed with the aim of providing both theoretical guidance and empirical evidence for the value co-creation governance practice of China’s PCSE.

## 2. Theoretical framework

### 2.1. Overview of public value theory

The ecosystem theory posits that members organise in the form of alliances, networks, and so forth, establishing a cooperative symbiotic relationship and thereby realising value co-creation in the promotion of common goals, products, or services [19, 20]. The subject of value co-creation in public cultural services encompasses the government, the entity responsible for the provision of public cultural services, and the public. The interaction and interdependence among the three parties involved in the process of achieving the public value goal facilitate the formation and evolution of the PCSE. The theory of public value offers an excellent analytical framework for elucidating the intricacies, resilience, and adaptability inherent to ecosystems.

The advent of new public service theory and new public governance can be attributed to a response to the shortcomings in the theoretical practice of new public management theory. However, this response has not addressed the generalisation of instrumental rationality and the absence of value rationality at its core. In 1995, Moore posited that the primary mission and objective of government is to achieve "public value", which necessitates the formulation of public policies and the provision of public services [21]. To integrate public value into strategic management within the public sector, Moore developed a novel analytical framework for administrative departments and managers known as the "strategic triangle model". The model is predicated on the questions of what constitutes public values and how they are created. The Public Value Strategic Triangle Model encompasses three dimensions: value, legitimacy, support, and operational capability. The fundamental logic of the model can be summarized as follows: firstly, it requires collaboration among multiple stakeholders to determine public values collectively while actively participating in establishing these values; secondly, it emphasizes ensuring legitimacy for defined goals related to public values by providing necessary policy support. This necessitates the clarification of the concept of public value through policy processes in order to establish a legal environment conducive to the co-creation of such values. Finally, by aligning internal operational processes with external environmental factors, the objective is to optimise resource allocation (including talent and technology) and improve operational capabilities within entities responsible for delivering public services, thus enabling the effective achievement of desired levels of public value. The strategic triangle model offers a clear and actionable framework for public managers to collaboratively generate public value, and has been extensively expanded upon and applied. For example, Cole & Parston (2006) introduced the Public Service Value Model (PSVM), which examines the definition of public outcomes and the quantification of public service value [22]. Subsequently, Moore (2013) developed a tool, the Public Value Scorecard, for the assessment of management strategy practices in the public sector [23]. Furthermore, in response to value conflicts and failures during the process of co-creating public value, Bozeman (2007) proposed consensus-led public values, which represent agreements on rights, obligations, and norms [24]. Jorgensen, Van Wart, Kernaghan, and other scholars have discussed various types and structures of public values from different perspectives, establishing an analytical paradigm known as the public values chain [25–27]. This provides a further specific operational guide for public managers in identifying public value.

### 2.2. Value co-creation governance framework of PCSE

#### 2.2.1 Framework of analysis

When policies and management strategies are politically legitimate, feasible and sustainable, and operationally feasible and practical, and when they have value to citizens, public value is created [21]. This constitutes a significant point of reference for the construction of a Public Cultural Services ecosystem in the context of the development of a service-oriented government in China. Furthermore, Van Der Wal & Van Hout (2009) argue that the diversity and conflict inherent in public values are primarily manifested through ambiguity, competing values, and hybrid organisations [28]. Consequently, achieving a harmonious balance between instrumental and value rationality [29] assists Chinese scholars and public managers in exploring the path towards local co-creation of public value in China. For instance, Men et al. developed an analytical framework based on the three-level model of public value strategy to facilitate Chinese government data openness [30]. In contrast, Shang et al. employed the theory of public value to elucidate issues pertaining to equalising Chinese public library services and realising value reproduction [31].

In the Chinese context, the PCS are run by the government and evaluated according to people’s satisfaction. The 14th Five-Year Plan for the Construction of PCSS explicitly states that the primary objective of public value should be to provide basic and equal PCS. In accordance with the Law of the People’s Republic of China on the Protection of PCS, as well as other pertinent legislation and government regulations, such as the Twelfth Five-Year Plan of the National Basic Public Service System, public cultural facilities, including public libraries, museums, memorial halls, art galleries, cultural centres, and science and technology centres, play a pivotal role in the delivery of these services, due to their inherent public attributes. Consequently, the objective of achieving public value with Chinese characteristics is regarded as a "completion state" [30]. In essence, the process of co-creation involves the Chinese government formulating policies and providing legitimacy and support, while public service carriers are responsible for specific operations with active participation from citizens. Consequently, it is necessary to localise the "strategic triangle model" in order to align it more closely with the value co-creation governance practice of China’s PCSE. Firstly, the "operational capability" in the original model is divided into two variables: "internal operational process" and "external co-creation environment". Secondly, the variables of "legitimacy and support" and "internal operation process" are combined to form a new variable, designated as "internal support and operation". Finally, the combination of these two new variables, along with the "public value goal", forms a novel framework for analysing public value co-creation: namely, "public value goal—internal support and operation—external co-creation environment".

#### 2.2.2. Strategic triangular relationship

In order to enhance the creation of public value, the carriers should establish a robust service community and ensure equal opportunities and equitable access to low-level PCS for all individuals, regardless of their needs or circumstances, through investments in infrastructure and resource allocation [32]. The achievement of the "public value goal" heavily relies on the carries, with the equalization level of basic PCS serving as an indicator for measuring public perception towards public value.

The process of co-creating value within China’s PCS sector exhibits distinct "Chinese characteristics". The government plays a leading role in this process by refining financial investment mechanisms and enhancing the guarantee Basic PCSS [14]. Consequently, the government provides "internal support and operation" for the value co-creation of PCS. Generally speaking, higher levels of GA correspond to increased support and assurance.

The public’s perception of public value significantly influences the service value orientation of carriers providing PCS. The "external co-creation environment" represents the safeguarding of the public’s fundamental cultural rights and is mutually dependent on their CN. This promotes the coordinated development of a modern PCSS and a modern cultural market system, meeting the diverse levels of CN in order to achieve the transformation of value co-creation outcomes.

**Fig 1:**
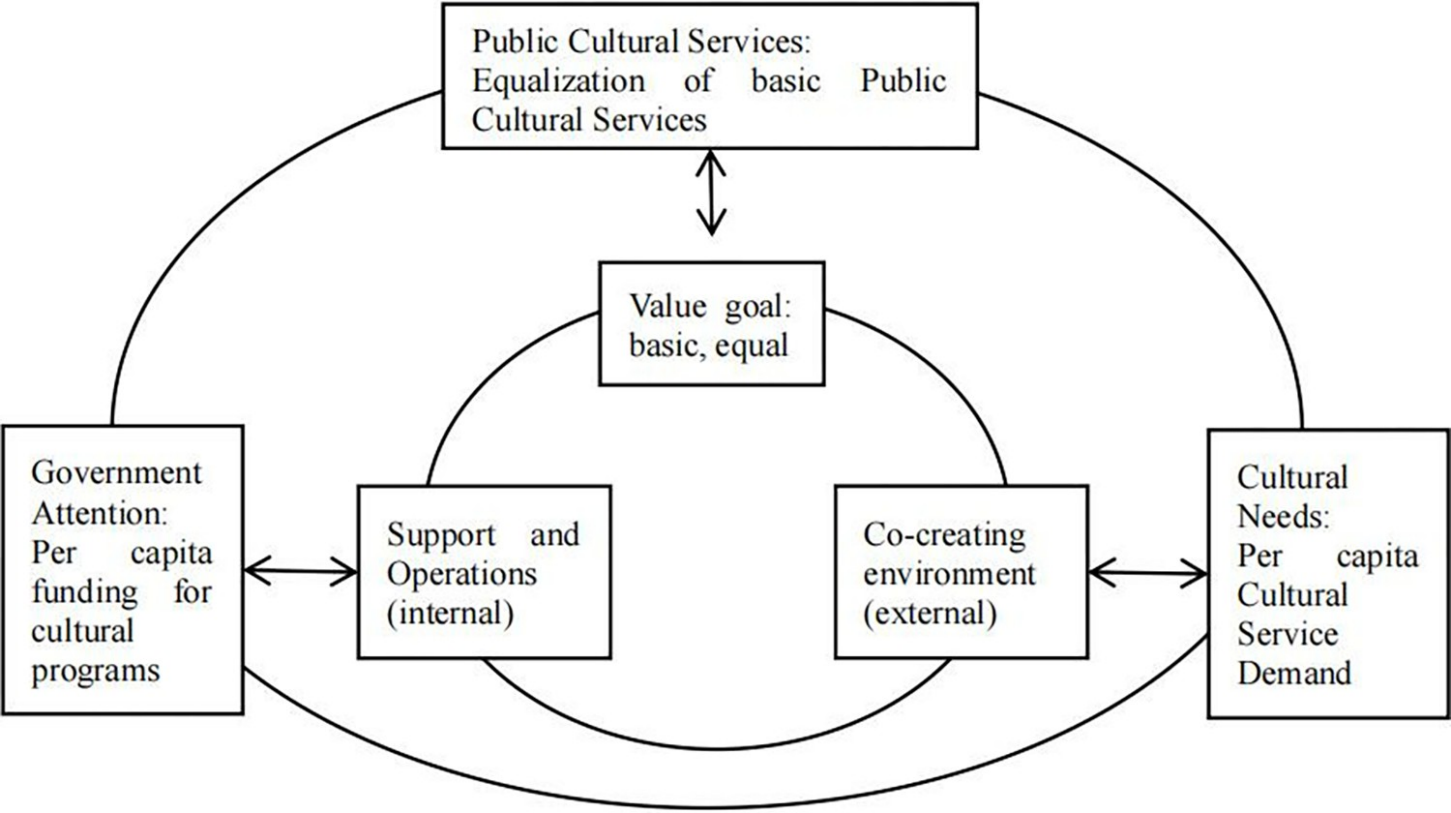
The strategic triangular relationship for the value co-creation of PCSE in China.

In summary, the strategic triangular relationship for the value co-creation of PCSE has further evolved into a relationship between GA, CN, and PCS. The process of value co-creation serves as an interaction mechanism among these three elements within the unified framework of the PCSE, as illustrated in Fig 1.

### 2.3. Mechanism of value co-creation

#### 2.3.1. GA and PCS

The primary objectives of the Chinese government in establishing a contemporary PCSE are to guarantee inclusivity and equal benefits for all [13]. In March 2014, the National Coordination Group on the Construction of the PCSS was formally established, with the Ministry of Culture at its helm and 25 departments in attendance, including the Propaganda Department of the Central Committee and the National Development and Reform Commission. Since the introduction of the concept of the "Public Cultural Services System (PCSS)" in 2005, the Chinese government has developed over 60 plans, laws, and regulations to promote standardisation and equalisation of basic PCS. These measures provide a robust foundation for establishing a modern PCSS. Conversely, China has been proactive in transforming its governmental functions, with a particular focus on strengthening its primary responsibility in providing basic PCS while fostering development momentum within this sector. From an initial appropriation budget of 13.382 billion yuan nationwide for cultural programmes in 2005, it is projected that this will rise to 120.289 billion yuan by 2022, representing an increase of approximately 898.89 percent. This substantial increase will provide robust support towards enhancing levels of PCS provision. As a subject with social public value attributes, governments are naturally inclined to direct their demands towards achieving social public value [31]. The existing literature [13–18] offers a theoretical explanation of the circular transmission relationship between GA and PCS, while also providing an empirical analysis of the "top-down" mechanism through which public value supply occurs.

#### 2.3.2. CN and PCS

It is widely acknowledged that public culture and business culture, as two major domains of CN in society, maintain a symbiotic relationship. Hu (2017) conducted an analysis from four perspectives: the evolutionary process, functional orientation, dynamic mechanism, and realistic conditions [15]. He argued that public culture and the cultural industry are mutually dependent and supported within the cultural ecological environment. Previous studies [10–12] have provided a comprehensive analysis of the internal logic and formation path of PCS, which act as a bridge for integrating and developing public culture with operational culture. It can be seen that the symbiotic equilibrium between the CN of the general public and government-led PCS establishes a "bottom-up" mechanism for transmitting public value demands.

#### 2.3.3. GA and CN

As the concept of "big culture" has become more deeply entrenched in Chinese society, the modern PCSE has established a fundamental construction mode that is characterised by "government-led, social participation". It strives to dismantle administrative barriers between departments in practice and aligns GA with the public’s CN. In the diversified construction, some local governments have tried to practice the new PCS model of the concept of public value co-creation, trying to open up the public value transmission mechanism of the two paths of "top-down" and "bottom-up". For example, in 2023, the Guangzhou Municipal Government explored the establishment of a "public cultural community" [33] and sought to establish an integrated exchange and cooperation mechanism that promotes co-construction and sharing of PCS. The objective of this initiative is to enhance the efficiency of the PCSS while establishing a new ecosystem for such services.

### 2.4 Research hypothesis

The existing literature on GA, CN and PCS is relatively mature, yet there remain several research gaps. (1) There is a paucity of research that incorporates GA, CN and PCS within a unified framework, with the majority of studies focusing on the one-way relationship between variables. (2) Model estimation may result in the omission of endogeneity and time delay among variables, leading to non-objective conclusions. (3) Without testing the dynamic interaction among variables, it is difficult to reveal the differences in different regions of China. In order to address the limitations of existing studies, we utilise the Public Value Strategic Triangle Model to construct a unified analytical framework and propose the following two research hypotheses.

H1: Within the same framework, there are dynamic interactions between GA and CN, GA and PCS, and CN and PCS.
H2: In the context of China’s spatial unbalanced development pattern, there are regional differences in the path of realisation of public cultural services.

A PVAR model has been constructed to examine the impact of initial inputs on other variables in the eastern and central regions. The Granger causality test, impulse effect diagram and variance decomposition results analysis have been employed to verify the long-term and short-term dynamic interaction effects among the three.

## 3. Research design

### 3.1. Entropy method

In order to measure the level of PCS in a more scientific way and to avoid any potential bias in the evaluation results, this study uses the entropy method, which is based on the approaches of scholars [18, 32], to calculate a comprehensive score that reflects the level of PCS. The calculation principle of the entropy method is as follows:

In the first step, since each secondary index has different dimensions and values that are widely dispersed, it is necessary to standardise the original data. As all secondary indicators in this study are positive indicators, the calculation formula of the same metric index y_ij_ is shown in Eq (1).


yij=[xij−MIN(xij)][MAX(xij)−MIN(xij)] (1)

The second step is to dimension the original index and calculate the same metric index p_ij_, as shown in Eq (2).


pij=xij∑j=1mxij (2)

In the second step, the entropy value of each second-level index is calculated e_i_, m is the number of samples, as shown in Eq (3).


ei=−k∑j=1n(pij∙lnpij),k=−1lnm (3)

The fourth step is to calculate the difference coefficient of each second-level index g_i_, as shown in Eq (4).


$$g_{i}=1-e_{i}$$ (4)

The fifth step is to calculate the weight of each second-level indicator w_ij_ and calculate the score, as shown in Eq (5).


wij=gi∑j=1mgi,Fi=∑j=1m(wij∙pij) (5)

### 3.2. PVAR model

The utilisation of brief panel data in this paper permits the revelation of the dynamic relationship between GA, CN and PCS under the same analytical framework. The panel vector autoregressive (PVAR) model, proposed by Holtz Eakin et al. (1988) [34], does not necessitate the existence of a pre-established causal relationship between variables. Instead, each variable is regarded as endogenous, and its influence on other variables within the model is analysed in conjunction with its lagged values. This approach is particularly suited to the analysis of large cross-sectional datasets with short time series.

Given the potential for spatial disparities in China, this study aims to construct three PVAR models at both national and regional levels. This will enable the heterogeneity of their dynamic relationships to be unveiled in a scientifically and accurately manner. Taking into account the spatial imbalance pattern of China’s economy, this study divides the 31 provinces and cities into two categories: the eastern developed region, consisting of 11 regions, and the central-western regions, consisting of 20 regions. The eastern developed region exhibits higher economic development and population proportion, as well as distinct variations in GA, public CN, and PCS compared to those observed in the central-western regions. The PVAR model is shown in Eq (6).


$$X_{it}=A_{0}+\sum_{1}^{p}A_{j}X_{it-j}+f_{i}+d_{i}+e_{it}$$ (6)

A_j_ is the coefficient matrix (j = 1,2,⋯,p) of the vector X_t−j_, p is the lag order, i indicates the number of provinces and municipalities at different levels, t represents for 2008 to 2022, f_i_ is the fixed effect, d_i_ is the sum time effect, and e_it_ is the random disturbance term.

The vector X_t−j_, as depicted in Eq (7), encompasses the following:

$$X_{it}=\left\{ \begin{array}{c} {lnGA}_{it}\\ {lnCN}_{it}\\ {PCS}_{it} \end{array}\right.$$ (7)

## 4. Data and variable description

### 4.1 Data description

The research samples are drawn from the provincial and municipal panel data of China from 2008 to 2022 (31 provinces and municipalities in mainland China, excluding Hong Kong, Macao and Taiwan). In order to ensure the testability of the research, the data in this paper are all from the Statistical Yearbook of Chinese Culture and Cultural Relics and the Statistical Yearbook of Chinese Culture and Related Industries. In order to facilitate the reproduction of the empirical analysis presented in this paper, the data used in the analysis are attached as Supporting Information, as detailed in S1–S3 Files.

### 4.2 Variable description

#### 4.2.1. Government attention (GA)

The concept of GA refers to the level of importance that the government places on the PCS. This reflects the aspect of "internal support and operation" in the value co-creation of PCS. Given the significant disparities in economic strength and resident population across different regions, per capita government funding for cultural undertakings is chosen as an indicator to represent the government’s concern for public cultural endeavors. In general, a higher degree of governmental emphasis on culture is associated with increased per capita investment in cultural undertakings. This study employs the formula "per capita investment in cultural undertakings = funding for cultural undertakings/resident population" for calculation purposes, with subsequent natural logarithm transformation denoted as lnGA, in accordance with the practices set out in [17, 18].

#### 4.2.2. Cultural Needs (CN)

The concept of CN refers to the multi-level and diversified demand of the public for cultural services, which reflects the influence of the "external co-creation environment" in the value co-creation of PCS. In general, a higher level of CN among the public is indicative of a greater requirement for PCS. In accordance with the methodological approach proposed by [12], this study employs per capita cultural and entertainment consumption expenditure as an indicator of per capita cultural service demand, with the objective of capturing fluctuations in CN among the public. Take the natural logarithm to represent the CN variable and write it as lnCN.

#### 4.2.3. Public cultural services (PCS)

The provision of PCS, as a non-profit resource allocation activity based on social benefits, aims to offer non-competitive and non-exclusive cultural products for the society [35]. It distinctly differs from operational culture and possesses the characteristics of public goods. The essence and direction of basic PCS are determined by public culture [36], reflecting the collective value orientation it holds [31]. This component plays a crucial role in our country’s modern PCS. In 2021, the "14th Five-Year Plan" for the Construction of PCSS emphasized comprehensive promotion of standardization and equality in basic PCS. Equalization refers to ensuring fair and accessible access to roughly equal basic PCS for the public, with equal opportunity at its core [32]. The equalization level of basic PCS reflects the creation of public value, making it suitable to represent the variable "public value goal" in the value co-creation of PCS. This paper employs entropy method along with Eqs (1–5) to comprehensively evaluate the equalization level of basic PCS, which is used to represent the variables of PCS and recorded as PCS.

## 5. Empirical analysis

### 5.1. Calculation results of entropy method

The equalization of PCS reflects the low level of undifferentiated demand and equality of opportunity for the PCS. According to scholars [17–18, 32], this paper selects three primary indicators and 24 secondary indicators to evaluate using the entropy method with Eqs (1–5). The results are presented in Table 1. The weight values calculated by the entropy method reasonably reflect the contribution of each secondary index to the equalization level of basic PCS. The total weight assigned to the first-level indicator service effect (C), which represents value rationality, is 68.09%. This demonstrates the value rationality inherent in China’s current construction of a modern PCSE, which is consistent with the concept of serving the people and better enables the realisation of public value goals.

**Table 1:** Evaluation system of equalization level of basic PCS.

First-level indicator	Weight	Second-level indicator	Serial number	Weight
Service Community (A)	14.72%	number of public libraries per 10,000 people	A1	2.05%
		number of performing arts organizations per 10,000 people	A2	5.24%
		number of arts performance venues per 10,000 people	A3	2.63%
		number of mass cultural institutions per 10,000 people	A4	2.46%
		number of museum institutions per 10,000 people	A5	2.34%
Basic Resources (B)	17.19%	public library holdings per capita	B1	2.67%
		per capita book purchase fee	B2	4.00%
		building area of public libraries per 10,000 people	B3	2.71%
		number of electronic reading room terminals per 10,000 people in public libraries	B4	2.18%
		number of public library employees per 10,000 people	B5	1.78%
		number of employees in mass cultural institutions per 10,000 people	B6	1.62%
		number of museum employees per 10,000 people	B7	2.23%
Service Effect (C)	68.09%	circulation of public libraries per 10,000 people	C1	4.80%
		lecture attendance per 10,000 public libraries	C2	9.96%
		exhibition visits per 10,000 public libraries	C3	5.58%
		training attendance per 10,000 people in public libraries	C4	6.69%
		domestic performances by art performing groups per 10,000 people	C5	6.21%
		art performance groups domestic performances per 10,000 spectators	C6	6.02%
		arts performance venues arts performances per 10,000 people	C7	4.80%
		audience per 10,000 at art performance venues	C8	5.05%
		art performance venues art performance per 10,000 spectators	C9	4.59%
		number of cultural activities organized by mass cultural institutions per 10,000 people	C10	4.74%
		museum training per 10,000 people	C11	6.13%
		museum visits per 10,000 people	C12	3.51%

### 5.2 Empirical results and analysis of the PVAR model

This paper uses Stata16 software and Dr. Lian Yujun’s pvar2 command [37] to estimate the PVAR model shown in Eqs (6–7).

#### 5.2.1 Stationarity test

The PVAR model necessitates rigorous data stationarity requirements, which this paper conducts the stationarity test on panel data in two steps to avoid the problem of "spurious regression".

In the first step, each variable is individually subjected to unit root test. This study employs the most rigorous unit root test, which incorporates both a linear time trend term and an individual fixed effect term. Table 2 presents the lagged one-period results of the Levin-Lin-Chu (LLC) and ADF-Fisher (ADF) unit root test. The results of the test indicate that, following first-order difference processing, the LLC and ADF statistics of Government Attention (dlnNA), Cultural Needs (dlnCN) and Public Cultural Services (dPCS) can all be considered to be stationary at the significance level of 5%. Consequently, the outcomes of the unit root test indicate that this group of variables exhibits stationarity following first differencing.

**Table 2:** T-value results of unit root test.

Variables	Type	Nation	Eastern	Central-Western
dlnNA	LLC	-23.513***	-14.735**	-7.755***
	ADF	-15.030***	-8.879***	-18.057***
dlnCN	LLC	-19.269***	-9.936**	5.115***
	ADF	-5.141***	-3.090***	-15.711***
dPCS	LLC	-30.005***	-20.385***	-2.597***
	ADF	-20.757***	-15.204***	-22.571***

Note:(1)

"*","**" and "***"indicate significance at the confidence levels of 1%, 5% and 10%, respectively. (2) "ln." represents the log value of the original data of the variable, and "d." represents the first-order difference. The meanings provided below shall remain unchanged unless otherwise specified.

In the second step, a cointegration test is conducted on the groups of variables to determine whether there exists a long-term equilibrium relationship among them. The Westerlund test can be employed to examine both homogeneity and heterogeneity of variables, including linear time trend terms and individual fixed effect terms (Type I), as well as only the individual fixed effect term (Type II). Table 3 presents the results of four cointegration test with a one-period lag. The results of the significance tests indicate that the existence of a cointegration relationship between GA, CN, and PCS. This implies a long-term dynamic equilibrium relationship among these three factors.

**Table 3:** Results of the cointegration test.

Westerlund test	Type	Nation	Eastern	Central-Western
homogeneity	I	-0.957	-2.078**	-0.472
	II	-2.501***	-2.105**	-1.636*
heterogeneity	I	-2.754***	-1.075	-1.811**
	II	-3.552***	-2.403***	-2.419***

#### 5.2.2 PVAR model estimated By GMM

In this paper, the forward In this paper, the forward helmert method is used to eliminate individual fixed effects and the mean method is used to eliminate time fixed effects. Then, the optimal lag periods of the national level, the eastern region and the central and western region are calculated respectively. The determination of the optimal lag period is based on the principle that the minimum values of the AIC, HQIC, and SBIC must exceed two. The results are presented in Table 4. The findings indicate that at the national level, five periods represent the optimal lag period. In contrast, three periods are identified as the optimal lag period for the eastern region, while four periods are identified as the optimal lag period for the central-western regions. The varying optimal lag periods across models demonstrate significant heterogeneity among China’s 31 provinces and cities. Consequently, further investigation into this heterogeneity is required, which necessitates dividing them into the eastern region as well as the central-western regions.

**Table 4:** The results of the optimal lag period.

Statistic	Nation	Eastern	Central-Western
Lag	5	3	4
AIC	-6.682*	-6.024*	-4.848
HQIC	-4.726	-4.551*	-6.046*
SBIC	-5.894*	-5.427*	-6.866*

Note

"*" indicates the optimal lag period under this information criterion.

Finally, GMM estimation is carried out according to the optimal lag period. The results of the PVAR model are shown in Table 5.The study found that PCS exert a significant influence on GA at the national level, as well as in the eastern, central-western regions. This influence was observed with a confidence level of 1%. The findings indicate that increased GA to cultural services leads to a rise in public demand for culture, which holds true across all regions. The impact of GA on PCS exhibits noticeable regional variations.

**Table 5:** GMM estimation results.

Explained Variable	Explanatory variable	Nation L5.	Eastern L3.	Central-Western L4.
		Coef. (P)	Coef. (P)	Coef. (P)
**h_dlnNA**	h_dlnNA	1.53(0.125)	1.43(0.151)	1.64(0.100*)
	h_dlnCN	2.64(0.008***)	0.67(0.505)	2.07(0.038**)
	h_dPCS	3.82(0.000***)	3.06(0.002***)	3.93(0.000***)
**h_dlnCN**	h_dlnNA	3.32(0.001***)	1.73(0.084*)	2.24(0.025**)
	h_dllnCN	-1.28(0.202)	-1.06(0.287)	-1.37(0.169)
	h_dPCS	-2.69(0.007***)	-0.33(0.744)	-1.78(0.074*)
**h_dPCS**	h_dlnNA	2.82(0.005***)	-0.31(0.756)	0.96(0.335)
	h_dlnCN	-0.82(0.410)	-0.13(0.899)	-0.25(0.803)
	h_dPCS	-1.86(0.063*)	-3.57(0.000***)	-0.65(0.515)

Notes: (1) "h" denotes the result of eliminating the fixed effect by the Helmert transformation. (2) "L1." means the lag of the first order. (3) The p-values are indicated in brackets.

The dependent variable in this study is GA. The study found that PCS exert a significant influence on GA at the national level, as well as in the eastern, central-western regions. This influence was observed with a confidence level of 1%. This suggests that an expansion of government-led PCS will further intensify the government’s attention, thereby fostering a virtuous circle of public value creation. However, in the eastern region, there is no statistically significant impact of CN on GA. This indicates that, from a national perspective and in the central-western regions, the public’s demand for cultural services has a direct influence on the government’s emphasis on providing PCS.

CN as the explained variable. The findings indicate that increased GA to cultural services leads to a rise in public demand for culture, which holds true across all regions. Notably, at the national level, this relationship is statistically significant at a 1% significance level and exhibits the highest magnitude of influence. This suggests that as the concept of cultural confidence advocated by the central government becomes deeply ingrained in people’s hearts, there is an increasingly evident recognition and respect for traditional culture and ideological values. In the long term, there is a negative correlation between PCS and CN. At both the national and central-western regional levels, this negative relationship reaches statistical significance at 1% and 10%, respectively. This indicates that government-provided PCS may partially displace public demand for culture. However, this effect is not pronounced in the eastern region.

PCS as the explained variable. The findings indicate that the impact of GA on PCS exhibits noticeable regional variations. Specifically, only the national level effect remains significant after a lag of five periods. While CN exert some inhibitory influence on PCS, this effect is not statistically significant across all regions. These results highlight the substantial path-dependent nature of PCS and its resistance to short-term influences from public cultural demands.

#### 5.2.3 Granger causality test

The panel data show stationarity for both sets of variables, as indicated by the results of the unit root and cointegration test. Consequently, within a unified framework, the Granger causality test can be applied to further elucidate the causal relationship between the three variables. The results are presented in Table 6.

**Table 6:** The results of Granger causality test.

Explained Variable	Explanatory variable	Nation L5.	Eastern L3.	Central-Western L4.
		Coef. (P)	Coef. (P)	Coef. (P)
h_dlnNA	h_dlnCN	28.193(0.000***)	10.538(0.015**)	4.358(0.360)
	h_dPCS	21.058(0.001***)	9.805(0.020**)	1.223(0.874)
	ALL	55.427(0.000***)	26.650(0.000***)	7.613(0.472)
h_dlnCN	h_dlnNA	54.796(0.000***)	7.839(0.049**)	35.765(0.000***)
	h_dPCS	10.989(0.052**)	2.832(0.418)	12.551(0.014**)
	ALL	78.777(0.000***)	8.929(0.178)	51.499(0.000***)
h_dPCS	h_dlnNA	13.179(0.022**)	6.448(0.092*)	8.688(0.069*)
	h_dlnCN	4.243(0.515)	4.704(0.195)	4.095(0.393)
	ALL	18.939(0.041**)	9.499(0.147)	9.095(0.334)

From the significance results at the national level. With a lag of 5 periods, we observe a statistically significant two-way Granger causality between GA and CN at the 1% confidence level, another two-way Granger causality between GA and PCS at the 5% confidence level, and a one-way Granger causality from CN at the 10% confidence level. These results are consistent with the above conclusions from our PVAR model analysis. First, proactive government initiatives to preserve cultural heritage and lead social cultural development play an exemplary role in society by stimulating a renewed pursuit of cultural services across all segments of society. This leadership not only promotes increased demand for PCS, but also encourages the consumption of for-profit cultural services, thereby contributing to the establishment of a distinctive Chinese system of cultural consumption and inheritance. Second, both GA and CN contribute significantly to meeting basic public CN. This underscores the successful implementation of China’s value co-creation concept for PCSE, which is led by the government with active participation from society.

Heterogeneity analysis is performed based on the significance results of the different regions. The results of the Granger causality tests for the eastern region and the national level are largely consistent. However, there are three main differences: first, the statistical significance of the results is significantly lower compared to the national level; second, the rejection probability value for PCS in relation to GA is 9.2%, indicating a weaker impact of improving PCS on GA in eastern China compared to the national level; third, no significant statistical value is found in the Granger causality test between CN and PCS. This may be due to the fact that commercial cultural services in the eastern region to some extent crowd out basic public CN. In contrast to the eastern region, the test results from the central-western regions show clear differences: firstly, GA does not significantly influence either CN or basic PCS; secondly, while CN may influence PCS in these regions, it appears that the public’s consumption of commercial cultural services does not fully satisfy their CN due to relatively low consumption levels, necessitating complementary provision by PCS.

In summary, Granger causality test shows that the triangular cyclic relationship between GA, CN and PCS is verified. However, since the granger between PCS and CN has no statistical significance, the value co-creation triangular cycle of PCSE is still inadequate in the actual operation.

### 5.3 Impulse response

**Fig 2:**
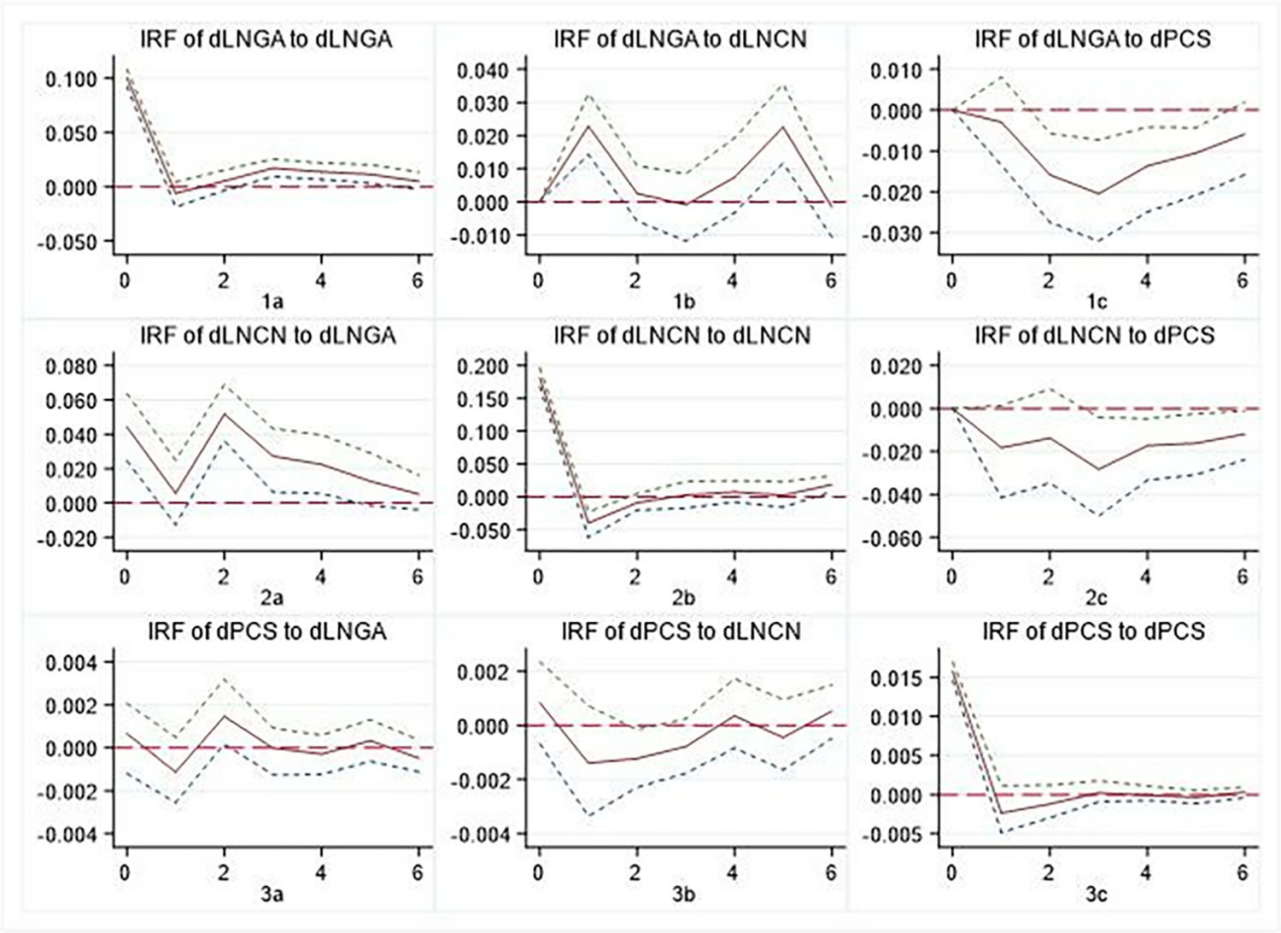
Impulse response of GA, CN, and PCS at the national. Notes: The horizontal axis represents the number of lag periods (years), the middle curve is the impulse response function curve. Errors are 5% on each side generated by Monte-Carlo with 200 reps. Same as below.

**Fig 3:**
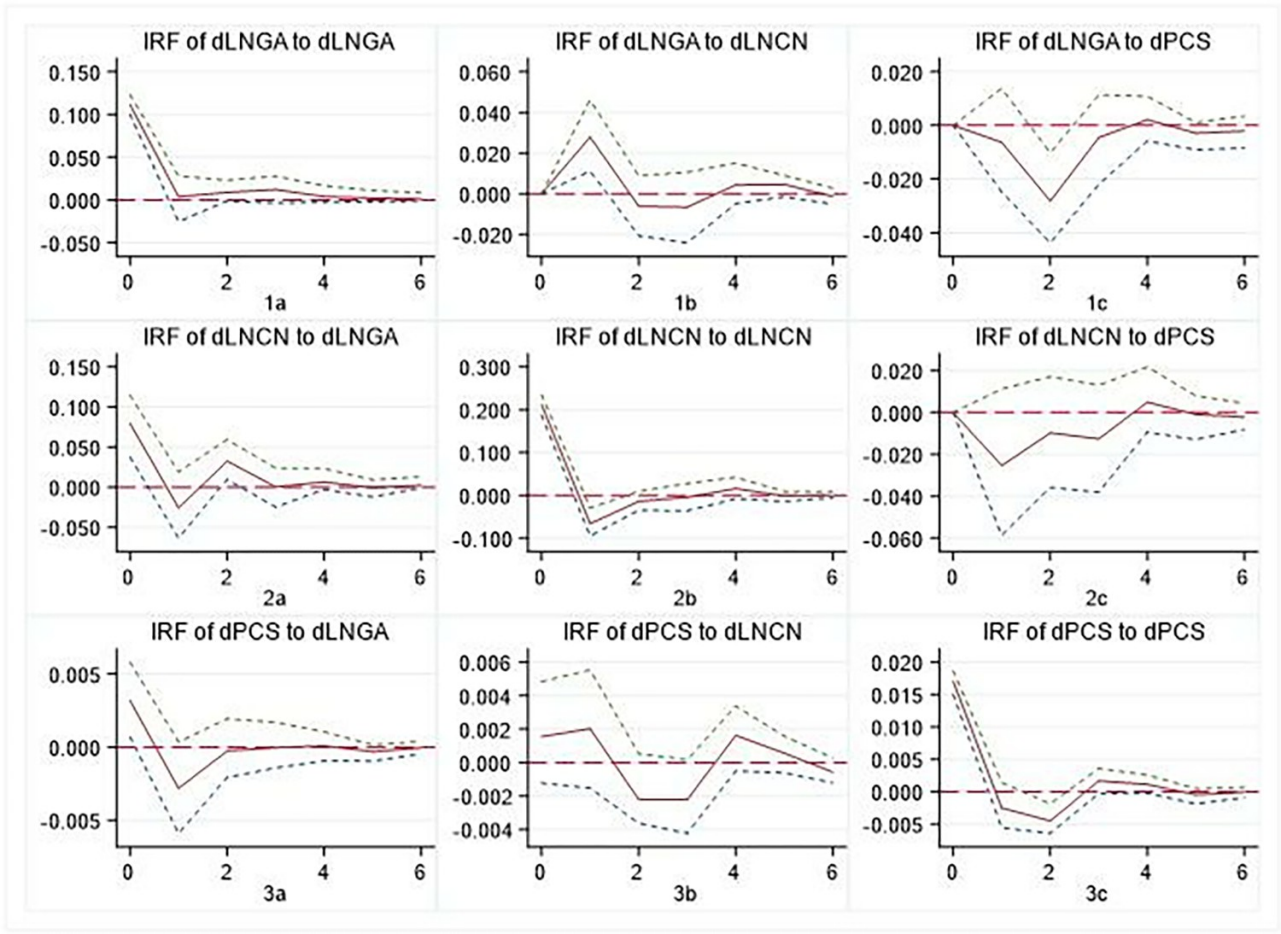
Impulse response of GA, CN, and PCS at the eastern.

**Fig 4:**
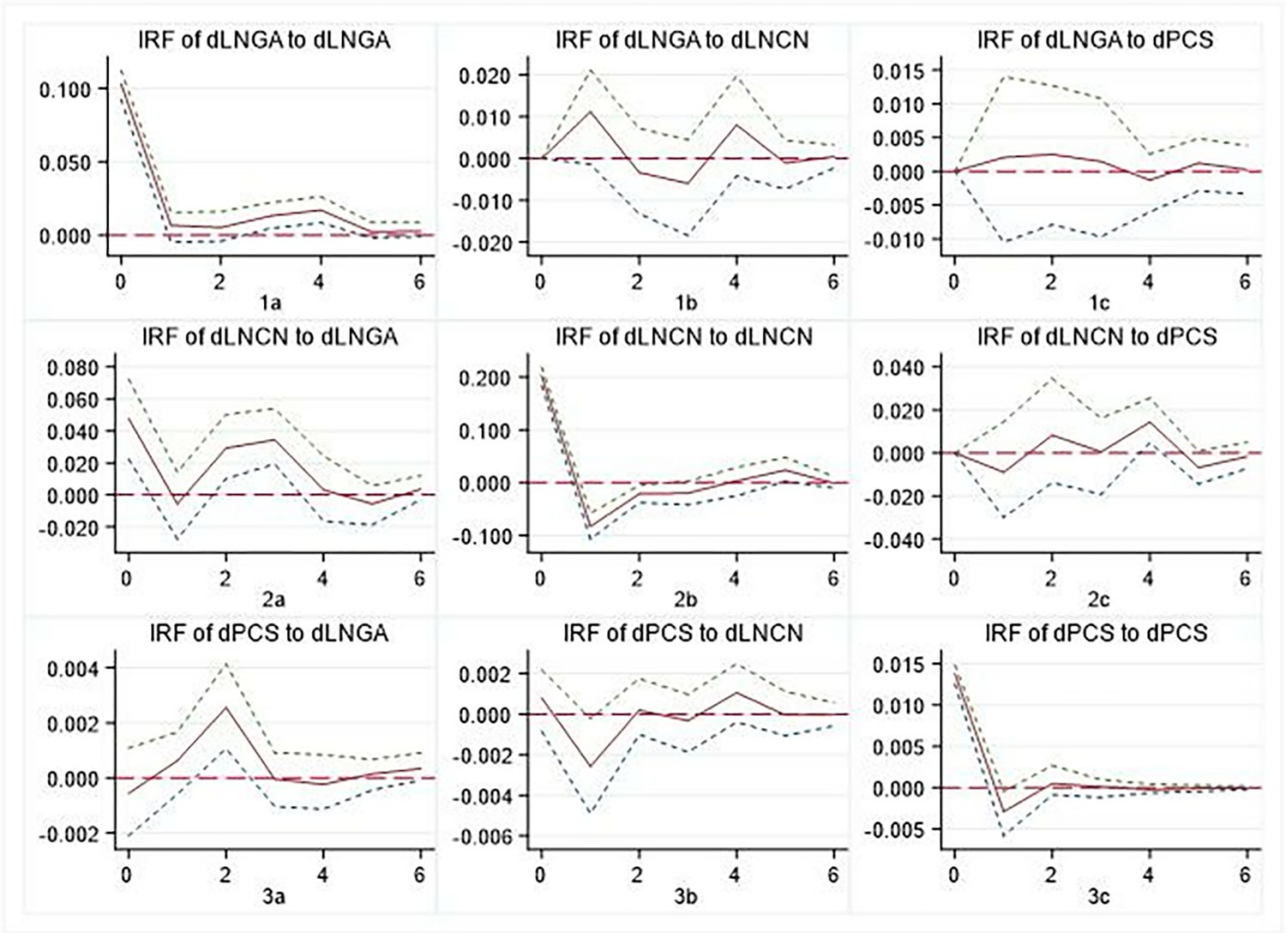
Impulse response of GA, CN, and PCS at the central-western.

The results of the impulse response function suggest that these relationships tend to stabilise by the sixth period in the future. Therefore, our analysis focuses on examining the dynamic changes of groups of variables over the next six periods, as shown in Figs 2–4. These figures show that both regions show similar patterns to those observed at the national level, albeit with some heterogeneity. It should be noted that after the first-order difference of panel data, the meaning of the variable represented is the newly augmented state, which is different from the meaning of the cumulative state represented by the original data. However, it does not affect the causal analysis between variables.

#### 5.3.1. GA and newly PCS

It is evident that there are discernible regional disparities in the interplay between newly augmented governmental attention and newly PCS. A comparison of Figs 2–4 reveals that there are different patterns between 1c and 3a. In contrast to the national level and the eastern region, the central-western regions demonstrate a significant mutual positive promotion relationship between GA and newly PCS. The response of newly PCS to the impact of 1 unit GA indicates that an increase in GA may, to some extent, impede the provision of PCS. Conversely, the introduction of 1 unit newly PCS will also result in a reduction in the government’s inclination to direct greater attention to cultural undertakings.

#### 5.3.2 CN and PCS

In the short term, the CN will partly impede the provision of PCS. The impact of PCS on CN varies significantly across regions, with the most pronounced repetition observed in the Eastern region. This can be seen from the difference between 2c and 3b, as shown in Figs 2–4. Firstly, there is a certain ’crowding out’ effect of for-profit cultural consumption on PCS, particularly evident in the Eastern region. Second, when making decisions about cultural consumption, individuals consider commercial cultural services as complementary to PCS; however, this complementarity varies across regions, resulting in different impulse response patterns.

#### 5.3.3 GA and CN

The GA and the CN both show positive and repeated interaction. Comparing 1b and 2a in Figs 2–4, we can see that First, there are variations in the degree of response, speed and repeatability of CN under the influence of 1 unit GA. Secondly, following the impact of 1 unit CN, GA shows positive effects, but their attenuation and recovery processes differ. This shows that despite changes in the structure of public consumption of cultural services, GA remains crucial for managing cultural inheritance and industrialization.

### 5.4 Variance decomposition

The important information of the random disturbance term can be further explored through variance decomposition analysis based on impulse response functions. Table 7 shows the changes in the proportion of impacts caused by shocks at both national and regional levels, distinguishing between two types of regional groups of variables.

**Table 7:** The results of decomposition of variance.

		Nation			Eastern			Central-Western		
	Period	dlnNA	dlnCN	dPCS	dlnNA	dlnCN	dPCS	dlnNA	dlnCN	dPCS
dlnNA	3	0.928	0.048	0.024	0.884	0.057	0.059	0.987	0.012	0.001
dlnCN	3	0.118	0.869	0.013	0.138	0.849	0.013	0.062	0.936	0.003
dPCS	3	0.015	0.016	0.970	0.052	0.033	0.915	0.034	0.034	0.932
dlnNA	6	0.839	0.085	0.076	0.878	0.062	0.060	0.978	0.021	0.001
dlnCN	6	0.143	0.813	0.044	0.138	0.847	0.016	0.082	0.911	0.007
dPCS	6	0.015	0.019	0.966	0.051	0.054	0.896	0.034	0.040	0.926

Overall, among the three variables, PCS have the highest contribution rate and the slowest decay rate. The bidirectional promotion effect among these variables is weak, while there are noticeable regional differences in the contribution rates of PCS to GA and CN. This clearly indicates that the diverse cultural consumption demands of Chinese citizens determine a long-term and extensive interrelationship between GA, CN and PCS.

From the perspective of the interaction between GA, CN, and GA. Firstly, the contribution rate of GA to PCS in various regions of China is relatively low, and has stabilized in the third period. The eastern region has the greatest impact, with a contribution rate of only 5.2% in the sixth period. Nevertheless, when compared horizontally, the figure is 3.4 times that of the national level and 1.5 times that of the central-western regions. Secondly, the contribution rate of CN to newly augmented newly augmented is essentially equivalent to that of GA. Similarly, the ability of the carriers to provide basic PCS in the eastern region is most susceptible to the influence of the government’s emphasis on culture and the public’s need for culture. Thirdly, the contribution rate of GA to CN is significantly higher than that of PCS. The contribution rate of CN to GA is also significantly higher than that of PCS. Fourthly, the contribution rate of PCS to GA and CN has significant regional differences.

From the perspective of the value co-creation triangular cycle of PCSE, the interaction between GA and CN, and the interaction between GA and PCS is relatively good, while the interaction between the CN and PCS is relatively weak. This is most evident in the central-western regions, where the contribution of PCS to GA and CN can be overlooked. And this demonstrates that both the national level and regional heterogeneity reflect the contradiction between the Chinese people’s growing demand for high-quality cultural consumption and the unbalanced and inadequate development that has occurred.

## 6 Research conclusions and policy suggestions

### 6.1 Research conclusions

To gain a comprehensive understanding of the value co-creation logic of PCSE in China, it is essential to analyse the key influencing factors within a unified framework. This paper not only deeply analyses the logic of value co-creation of PCSE in China under the necessity state, but also constructs a theoretical analysis framework based on the public value strategic triangle model, and studies the mechanism of value co-creation under the realistic situation. Using the panel data of mainland China from 2008 to 2022, this paper constructs a PVAR model from a dynamic endogenous perspective to empirically test the interaction and regional heterogeneity between Government Attention (GA), Cultural Needs (CN) and Public Cultural Services (PCS). The specific findings are as follows:

The results of the PVAR model and Granger causality test have been verified and analysed alongside the findings from impulse response and variance decomposition, leading to a similar conclusion. That is, the empirical results show that Hypotheses 1–2 are all valid. Overall, there exists a significant positive mutual promotion relationship between GA and PCS. In contrast, the two-way promotion relationship between CN and PCS is weak, while the relationship between GA and PCS is unstable. These empirical research findings provide strong support for the theoretical analysis framework based on the Public Value Strategic Triangle Model, thereby addressing the lack of empirical research in previous studies [13–16]. This paper integrates GA, CN, and PCS into a unified analytical framework for public value co-creation, revealing their dynamic relationships that contribute to our understanding of the logic behind the value co-creation of PCSE in China.

The optimal lag period of the PVAR model at the national level is five periods, which is weak in timeliness, indicating that there are certain differences between regions. With regard to the impact of PCS, CN play a more prominent role than PCS in eastern China, highlighting the significance of "bottom-up" demand transmission mechanism for achieving public value goals when PCS are high. Conversely, the influence of GA on PCS is more pronounced in central-western regions than in eastern regions, highlighting the significance of the "top-down" supply transmission mechanism for co-creating public value when PCS are low. This innovative conclusion addresses the limitations of Liu [10] and Peng [12], reveals regional comparative advantages diversity in China, and provides empirical evidence for developing differentiated pathways towards co-creating values of PCSE.

### 6.2 Discussion

While this paper offers a valuable analysis of the interaction mechanism among the three subjects in the ecosystem, namely the government, the public cultural services carrier, and the public, it also provides significant supplementary evidence to existing studies. Nevertheless, the PVAR model’s intrinsic limitations preclude its capacity to elucidate the process of value co-creation realisation within the Public Cultural Services ecosystem. Furthermore, it is unable to fully elucidate the potential mediating effects between variables. Furthermore, the present paper does not sufficiently address the underlying causes of regional disparities, which will be elaborated upon in subsequent research. It is evident that the advent of new digital technologies and sustainability innovations is transforming the governance model of the Chinese government, consequently exerting a considerable influence on the Public Cultural Services ecosystem. The impact of technology and innovation on the mode and path of value co-creation of public cultural services will continue to be the subject of further study.

In light of the aforementioned findings, this study makes the following significant theoretical and practical contributions. Firstly, it is important to highlight that previous literature has primarily focused on exploring the one-way influence relationship between these factors. In contrast, this paper incorporates all three into a comprehensive framework for dynamic two-way research, representing a unique contribution to the field. This research empirically examines the strategic triangle relationship in achieving public value goals within the public cultural services ecosystem, thereby enhancing our understanding of the symbiotic relationship among behavioural agents. Secondly, the utilisation of panel data to establish a PVAR model can enhance the scientific rigour of research findings and facilitate a comprehensive understanding of the operational mechanisms within China’s current PCSE. Thirdly, from a practical standpoint, this study examines both national-level dynamics and regional disparities, thereby offering empirical evidence and policy entry points for the construction of an adaptable public cultural services ecosystem tailored to local conditions. The findings of this research are applicable not only to China but also to other countries and regions around the world. They provide guidance for governments seeking to establish localised public value co-creation public service ecosystems and to facilitate the transition towards a service-oriented government.

### 6.3 Policy suggestions

The present study proposes policy recommendations for enhancing the value co-creation governance practice of China’s PCSE, based on the aforementioned research findings.

At the national level, it is of the utmost importance for the government to expedite the establishment of a service-oriented administration and effectively implement value-based governance [38]. This necessitates the formulation of adaptable public value co-creation policies that consider both the "top-down" supply transmission path and the "bottom-up" demand transmission path, ensuring a virtuous cycle within the strategic triangle model of public cultural value. On the one hand, differentiated public value objectives should be formulated in light of variations in population structure, particularly regarding distinct demands for PCS between urban and rural residents. On the other hand, there is a necessity to accelerate the transition from the traditional PCSS to a digitalised one [39], utilising innovative digital technologies to provide equitable access to basic PCS across urban and rural areas.

In eastern China, a priority for the value co-creation of PCS should be the enhancement of the "bottom-up" demand transmission pathway, as well as the facilitation of the integration and development of CN and PCS. Given the relatively developed economy in the region, there is a heightened pursuit for high-quality CN among its residents, along with a strong willingness to actively participate in the value co-creation of PCS. Local governments should adopt the guiding principle of spiritual life’s common prosperity [40], effectively guide public values, explore a multi-stakeholder system for PCS, and enhance the overall quality of people’s spiritual and cultural lives [41]. For example, the release of Guangdong Province’s Guidelines for Building a Public Cultural Services Community in 2024 represents an exemplary endeavour.

The path of the value co-creation of PCS in the central-western regions should focus on the smooth "top-down" supply and transmission path, effectively implementing the series of plans for constructing a modern PCSE formulated at the national strategic level, and placing greater emphasis on cultural undertakings. The PCS in the central-western regions are relatively inadequate, failing to effectively meet the public’s CN. Consequently, it is essential for local governments to enhance the synergy among subjects involved in public cultural governance [42], coordinate investments in public culture, improve the balance of available cultural resources, standardize the quality of PCS, and enhance the ecosystem of value co-creation within public services [6].

## Supporting information

S1 File (CSV)

S2 File (CSV)

S3 File (CSV)

## References

[1] Jay H. (2023).A ’Public Service Internet’-Reclaiming the Public Service Mission. Political quarterly. doi: 10.1111/1467-923X.13337

[2] Shi X.H.(2010).Fundamental Meanings Theoretical Basis and Constructing Conditions of Service—Oriented Government. Journal of Social Sciences,(02):3–11+187. https://chn.oversea.cnki.net/KCMS/detail/detail.aspx?dbcode=CJFD&dbname=CJFD2010&filename=SHKX201002002&uniplatform=OVERSEA&v=QhT4ERd_RLhJ4jaNDJQHIZg04D_Rud0ZAvP592kYnmO102mZjbJP90zCO6JmcOP3

[3] Tian X.L. (2023). Post-NPM Movement and China’s SOG Reforms. Public Administration and Policy Review,12(02):157–168. https://chn.oversea.cnki.net/KCMS/detail/detail.aspx?dbcode=CJFD&dbname=CJFDLAST2023&filename=GGZC202302011&uniplatform=OVERSEA&v=T-TTKMBpzT2CPOH-zI73wlGapO-bQxwTEZiYYmFkBvDexm2EHDRVOeML35NOsiHS

[4] Cui X. & Tang W.Y. (2024). Research on the Symbiotic Ecology of In-depth Cooperation Between Industry, Academia and Research in Grassroots Libraries Under the Government’s Purchase of Public Cultural Services: Taking the Library of Beilin District, Xi’an as an Example. New Century Library, (01):5–11. doi: 10.16810/j.cnki.1672-514X.2024.01.001

[5] Trischler J., Rohnebaek M., Edvardsson B. & Tronvoll B. (2023). Advancing Public Service Logic: moving towards an ecosystemic framework for value creation in the public service context. Public management review. doi: 10.1080/14719037.2023.2229836

[6] Ojasalo J. & Kauppinen S. (2022). Public Value in Public Service Ecosystems. Journal of nonprofit & public sector marketing, 36(02):179–207. doi: 10.1080/10495142.2022.2133063

[7] Jin W.G., Su Y.L., Li Y. (2024). Public Cultural Community: A New Path of Modern Governance under the Perspective of "Broad Culture"-The Construction of Quality and Efficiency Measurement Models for Public Cultural Facilities. Library Tribune. https://chn.oversea.cnki.net/KCMS/detail/detail.aspx?dbcode=CAPJ&dbname=CAPJLAST&filename=TSGL20240124001&uniplatform=OVERSEA&v=doTgzj28lf4BPX0qCo25ZOUZ_RipfLRVbz0aEK3u0PEJaJ5cu3kqJFLS4NeHYZ6G

[8] Xiao P. & Zhang W.X. (2024). Towards a "Public Cultural Service Community": Innovative Exploration and Experience Promotion of Guangdong’s Public Cultural Service System. Library Tribune. https://chn.oversea.cnki.net/KCMS/detail/detail.aspx?dbcode=CAPJ&dbname=CAPJLAST&filename=TSGL20240318001&uniplatform=OVERSEA&v=doTgzj28lf7KOyT4Tg1gzoi06oifZVSNk3Ld2_yBzrHMr-c3LVUVyUjMiP0jJYbe

[9] Li S.H. & Yuan S. (2023). The symbiotic relationship between intangible cultural heritage resources and Public Cultural Services in western China and its influencing factors.Library Development. https://kns.cnki.net/kcms/detail/23.1331.G2.20230506.1058.004.html

[10] Liu Y. & Zhou J. X. (2020). Coordinated development of Public Cultural Services and cultural industry: An empirical study based on panel data from 31 provinces. Jiangxi Social Sciences, 40(03):72–84. https://chn.oversea.cnki.net/KCMS/detail/detail.aspx?dbcode=CJFD&dbname=CJFDLAST2020&filename=JXSH202003009&uniplatform=OVERSEA&v=yVn0jpPHuKBWVIXM3fgy3TtZwuegtC23xVvOPxh0BbBH3PcHxpkqHoKNRXgwM0TR

[11] Zhang H.F., Yang F.T., Jing Q.L. (2022). Public Cultural Services and High-Quality Economic Development: Policy Implications Based on Coupling Coordination Degree Model. Review of Economy and Management, 38(02): 58–70. doi: 10.13962/j.cnki.37-1486/f.2022.02.005

[12] Peng L.T. & Kang Y.M. (2023). Study on the Measurement of the Integration Level of Culture and Tourism in China:an Exploraton Based on the Coupling and Coordination of Public Cultural Services, Cultural Industry and Tourism Industry. Decision & Information, (06):47–59. https://chn.oversea.cnki.net/KCMS/detail/detail.aspx?dbcode=CJFD&dbname=CJFDLAST2023&filename=JCYX202306006&uniplatform=OVERSEA&v=qn-_kur1Ruwzb1BXgYSa3Ov2P2TQHukRyPpTqR34cBEE4LdoFUqitEiXA0VxacPb

[13] Li S. (2024). The Historical Logic, Chinese Characteristics, and Development Prospects of Modern Public Cultural Service System.

[14] Shang J.K. (2024). A Probe into the Construction of Equalization of Public Cultural Services in the New Journey of Chinese path to modernization.

[15] Hu S.Y. (2017). Fusion Development of Public Culture and Cultural Industry: Inner Logic, Realistic Predicament and Advancing Path.

[16] Tao J. & Chen Q.Q. (2023). High- quality Integrated Development of Public Cultural Services and Tourism. Library Journal,43(10):47–54.

[17] Gan D.J.(2023). Evaluation on Equalization of Urban and Rural Public Cultural Services and Its Index System Construction. Academic Journal of Zhongzhou,(12):77–85.

[18] Peng L.T. & Zhang L.(2023). Research on the Evaluation of High-quality Development of Public Cultural Services. Journal of Macro-quality Research, 11(02):90–101. doi: 10.13948/j.cnki.hgzlyj.2023.02.007

[19] Moore J. F. (1993). Predators and prey: a new ecology of competition. Harvard business review, 71(3), 75–86. 10126156

[20] Adner R. (2006). Match your innovation strategy to your innovation ecosystem. Harvard business review, 84(4), 98. 16579417

[21] Moore M.H. (1995). Creating Public Value: Strategic Management in Government.International Journal of Professional Business Review, 6(1):219. doi: 10.26668/businessreview/2021.v6i1.219

[22] Cole M. & Parston G. (2006). Unlocking Public Value:A New Model for Achieving High Performance in Public Service Or—ganizations. Hoboken,NJ:John Wiley & Sons. http://www.untag-smd.ac.id/files/Perpustakaan_Digital_2/PUBLIC%20ADMINISTRATION%20Unlocking%20public%20value%20%20a%20new%20model%20for%20achieving%20high%20performance%20in%20publi.pdf

[23] Moore. Mark H. (2013).Recognizing Public Value. Cambridge, MA: Harvard University Press. 10.1002/pam.21776

[24] Bozeman B. (2007).Public Values and Public Interest:Counter-balancing Economic Individualism. Washington, DC: Georgetown University Press. doi: 10.1353/book13027

[25] Jorgensen T.B. & Bozeman B. (2007). Public Values: An Inventory. Administration & Society,39(3):354–381. doi: 10.1177/0095399707300703

[26] Van Wart M. (1998). Changing Public Sector Values. New York: Routledge Press. doi: 10.4324/9780203054635

[27] Kernaghan K. (2003). Integrating Values into Public Service:The Values Statement as Centerpiece. Public Administration Review, 63(6):711–719. doi: 10.1111/1540-6210.00334

[28] Van Der Wal Z., Van Hout E.Th. J (2009). Is Public Value Pluralism Paramount? The Intrinsic Multiplicity and Hybridity of Public Values. International Journal of Public Administration, 32(3):220–231. 10.1080/01900690902732681

[29] Xu J.B., He L.T. & Chen H.H. (2020). Balancing instrumental rationality with value rationality: towards avoiding the pitfalls of the productivist ageing policy in the EU and the UK. European journal of ageing,17(02):251–257. doi: 10.1007/s10433-019-00527-9 32549876 PMC7292837

[30] Men L.X., Zhao Z.M., Li Y.L. & Zhang H.P. (2024). Public Value Co-Creation Logic, Current Situation and Optimization Path of Open Government Data in China:Based on the Public Value Strategic Triangle Model.Information Studies:Theory & Application,47(02):91–97+106. doi: 10.16353/j.cnki.1000-7490.2024.02.013

[31] Shang Z.J., Li Q. & Yang B. (2024). Research on the equalization of public library services based on the theory of "public value". Library. https://kns.cnki.net/kcms2/article/abstract?v=YoFA4grnCX5wYQF66mDiYZMYG10jSSCsGb8a8hzmhymol9i6QmHum5bgH5o2pIf4RwSd31hHvWobjpLDZXW0TZeFOerF3r8reiJd079wYmHYHHoWs0rUoyuCqqz9cm3-vaa75ffvU_A=&uniplatform=NZKPT&language=CHS

[32] Wang L.Z. & Li F (2013). Public Cultural Services in China: Construction of Index System and Measurement of Regional Disparity. Comparative Economic & Social Systems, (01):184–195. https://chn.oversea.cnki.net/KCMS/detail/detail.aspx?dbcode=CJFD&dbname=CJFD2013&filename=JJSH201301022&uniplatform=OVERSEA&v=5cz5-rGel_B-zYrHlp3AGIbHExGpj7HcNIkLJIdXkF3C52_5ScB1iXupn0H9_5Hj

[33] Xiao P. & Li H.E. (2023). Public Cultural Community:Theoretical Foundation,Practical Exploration,and Institutional Construction. Library Tribune, 43(12):116–121. https://chn.oversea.cnki.net/KCMS/detail/detail.aspx?dbcode=CJFD&dbname=CJFDLAST2024&filename=TSGL202312017&uniplatform=OVERSEA&v=YbIlMFAHZIvEla-qjCJtb3_ZMTbd-TSHG3yY5nbtWSdVfI3UjE5MgK-5oC8tjfF9

[34] Holtz-Eakin D., Newey W. & Rosen H.S. (1988). Estimating Vector Autoregressions with Panel Data. Econometrica, 56(06): 1371–1395. https://www.jstor.org/stable/1913103

[35] Zhou X.L. & Mao S.L. (2008). On the Public Cultur e Service and Model Choice in China. Jiangsu Social Sciences, (01):90–95. doi: 10.13858/j.cnki.cn32-1312/c.2008.01.007

[36] Ke P., Gong P. & Wei Y.X. (2015). Review of Research on Public Cultural Services in China. Journal of the National Library of China, 24 (02):10–17. doi: 10.13666/j.cnki.jnlc.2015.02.003

[37] Lian Y. J. (2009). Research on Investment Efficiency of listed companies in China. Economics and Management Press.

[38] Chen Jian. (2024). Xi Jinping Thought on Culture Opens Up A New Realm of Chinese Modernization of Public Cultural Services. Library Tribune. https://chn.oversea.cnki.net/KCMS/detail/detail.aspx?dbcode=CAPJ&dbname=CAPJLAST&filename=TSGL20240228001&uniplatform=OVERSEA&v=doTgzj28lf4poL6FQMojc6kaHqdRIG_YW5v1vg5kgKSSOr2uLsNid9335iV7Yw77

[39] Wanyan D.D. & Hu J.H. (2020). How to provide public digital cultural services in China? Library hi tech, 2020, 38 (03): 504–521. doi: 10.1108/LHT-03-2019-0071

[40] Huang Y.W. (2023). The Shift, Dilemma and Adaptation of Public Cultural Governance from the Perspective of Common Prosperity in Spiritual Life. Academic Journal of Zhongzhou, (12):70–76. https://chn.oversea.cnki.net/KCMS/detail/detail.aspx?dbcode=CJFD&dbname=CJFDLAST2023&filename=ZZXK202312009&uniplatform=OVERSEA&v=jTz8RvzVdCr3JeS3TpYO30GkUDsgN72i0vzPQp6Zgah-T6IEd1zXm3XlBtWxmSFC

[41] Bao C.Q. & Bai L. (2024). Publicity: The value expectation and order construction of people’s spiritual and cultural life in the new era. Ideological & Theoretical Education, (02):47–54. doi: 10.16075/j.cnki.cn31-1220/g4.2024.02.008

[42] Wang X.J. & Zhang H. (2013). Research Approaches and Cutting-edge Questions of Public Value(s). Journal of Public Management, 10(02):126–136+144. https://kns.cnki.net/kcms2/article/abstract?v=YoFA4grnCX5hgL9C5p282X9k5ROpcmoAu9S6PzvJPveaGinZf8hRc4_yzRhhNODH02uHnHynZ9IadhyFqPeXpNvomuwg4BQR5YT3tvXln-flkWaA_xBnsJOOHUdV0DymJw-fWjpIsBA=&uniplatform=NZKPT&language=CHS

